# Activation of 2′ 5′-Oligoadenylate Synthetase by Stem Loops at the 5′-End of the West Nile Virus Genome

**DOI:** 10.1371/journal.pone.0092545

**Published:** 2014-03-20

**Authors:** Soumya Deo, Trushar R. Patel, Edis Dzananovic, Evan P. Booy, Khalid Zeid, Kevin McEleney, Stephen E. Harding, Sean A. McKenna

**Affiliations:** 1 Department of Chemistry, University of Manitoba, Winnipeg, Manitoba, Canada; 2 National Centre for Macromolecular Hydrodynamics, University of Nottingham, Sutton Bonington, United Kingdom; 3 Department of Biochemistry and Medical Genetics, University of Manitoba, Winnipeg, Manitoba, Canada; 4 Manitoba Institute for Materials, University of Manitoba, Winnipeg, Manitoba, Canada; University of British Columbia, Canada

## Abstract

West Nile virus (WNV) has a positive sense RNA genome with conserved structural elements in the 5′ and 3′ -untranslated regions required for polyprotein production. Antiviral immunity to WNV is partially mediated through the production of a cluster of proteins known as the interferon stimulated genes (ISGs). The 2′ 5′-oligoadenylate synthetases (OAS) are key ISGs that help to amplify the innate immune response. Upon interaction with viral double stranded RNA, OAS enzymes become activated and enable the host cell to restrict viral propagation. Studies have linked mutations in the *OAS1* gene to increased susceptibility to WNV infection, highlighting the importance of OAS1 enzyme. Here we report that the region at the 5′-end of the WNV genome comprising both the 5′-UTR and initial coding region is capable of OAS1 activation *in vitro*. This region contains three RNA stem loops (SLI, SLII, and SLIII), whose relative contribution to OAS1 binding affinity and activation were investigated using electrophoretic mobility shift assays and enzyme kinetics experiments. Stem loop I, comprising nucleotides 1-73, is dispensable for maximum OAS1 activation, as a construct containing only SLII and SLIII was capable of enzymatic activation. Mutations to the RNA binding site of OAS1 confirmed the specificity of the interaction. The purity, monodispersity and homogeneity of the 5′-end (SLI/II/III) and OAS1 were evaluated using dynamic light scattering and analytical ultra-centrifugation. Solution conformations of both the 5′-end RNA of WNV and OAS1 were then elucidated using small-angle x-ray scattering. In the context of purified components *in vitro*, these data demonstrate the recognition of conserved secondary structural elements of the WNV genome by a member of the interferon-mediated innate immune response.

## Introduction

**Figure 1:**
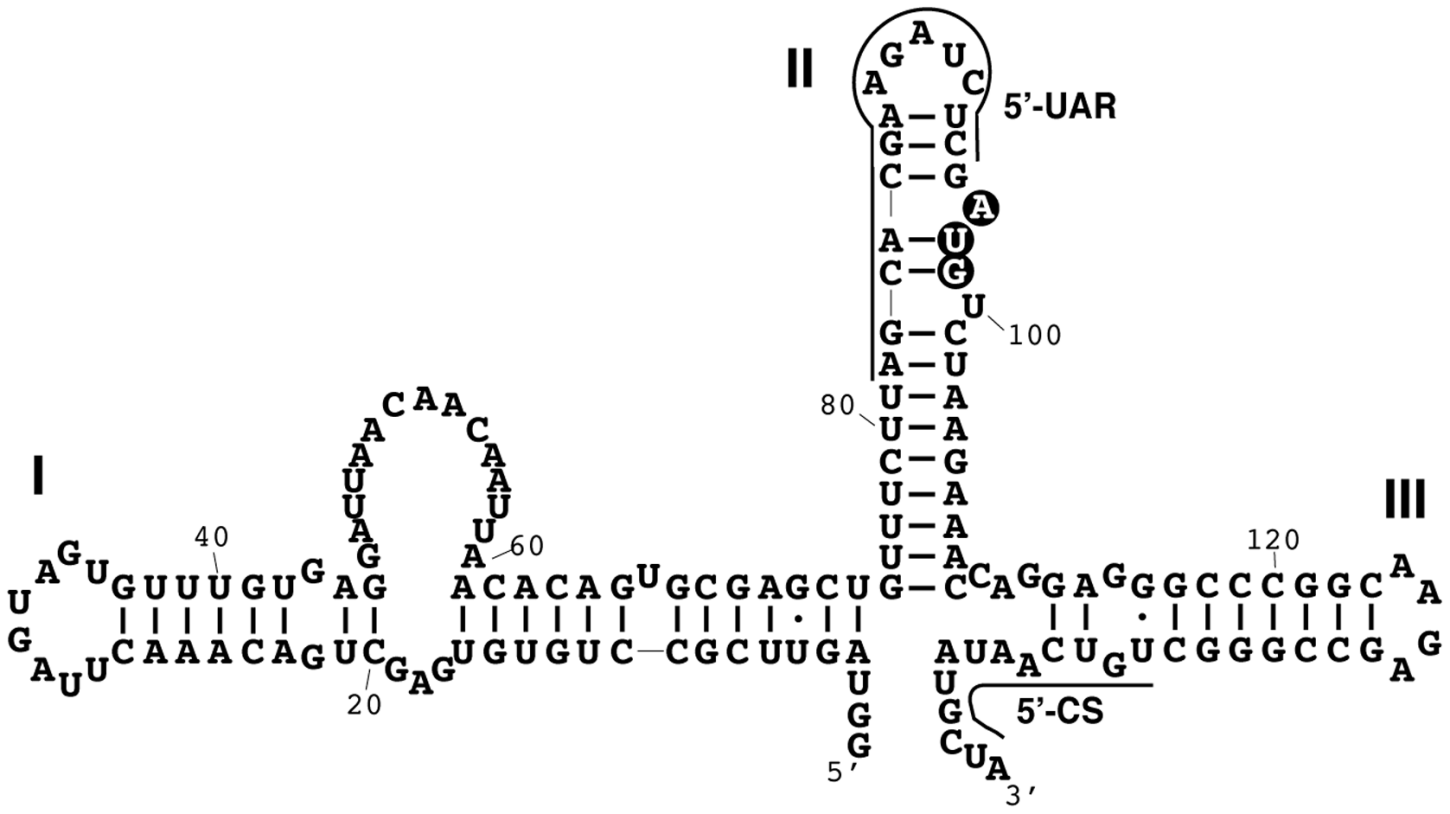
Secondary structure of the WNV 5′-end. Highlighted are SLI, SLII, SLIII, the AUG start codon (black circles), the upstream AUG region (SLI/II/III, solid line) and the conserved sequence element (5′-CS, solid line) [11].

West Nile virus (WNV) is an 11 kb positive-sense, single-stranded RNA virus that belongs to the *Flaviviridae* family. This family includes known pathogenic viruses that cause Yellow fever, Dengue fever, Japanese encephalitis, and Tick-borne encephalitis [1]–[5]. The viral genome consists of a single open reading frame (ORF) that encodes a large polyprotein precursor containing both structural and non-structural proteins [6]–[9]. The ORF is flanked upstream and downstream by the 5′ and 3′ -untranslated regions (UTRs) which, based on thermodynamic predictions, are rich in stable secondary structures and highly conserved amongst *Flaviviridae* family members despite the lack of extensive sequence homology [9]–[12]. Regions in both the 5′ and 3′-UTRs are necessary for translation initiation and minus strand RNA synthesis [13], [14]. The 5′-UTR and downstream initial coding region (SLI/II/III; nucleotides 1–146) is comprised of three stem-loops (SLI; nucleotides 1–73, SLII; nucleotides 73–110, and SLIII nucleotides 111–146), of which SLII contains the AUG start codon for polyprotein translation (**Fig. 1**). RNase probing experiments confirmed these predicted secondary structures [11]. Furthermore, WNV non-structural protein 5, a methyltransferase, binds specifically to the SLI of genomic RNA, and this structure is essential for viral RNA genome replication [11]. RNase probing of the dengue virus 5′-UTR regions demonstrates similar secondary structure interactions in SLA (equivalent to SLI in WNV), suggesting a common structural arrangement of the region amongst flavivirus family members [15], [16].

Two long distance interactions mediated by complementary base pairing between the 5′-UTR/initial coding region and 3′-UTR are thought to be required for WNV genome cyclization, creating a panhandle structure. Both the 5′-UAR (upstream initiation AUG region, in SLII) and 5′-CS (conserved sequence, in SLIII base pair with their complimentary sequence from regions in the 3′-UTR (3′-UAR and 3′-CS respectively) to achieve cyclization. Panhandle formation enables recruitment of factors, including the viral RNA dependent RNA polymerase [13], [14], [17], that are required for minus-strand RNA synthesis. Taken together, the structural features of the WNV SLI/II/III and other *Flaviviridae* family members represents a highly structured and important regulatory region.

The innate immune system is the first line of defense against viral infection, and susceptibility of IFN α/β receptor deficient mice to WNV infection points towards the importance of antiviral immunity conferred by the Type 1 IFN [18], [19]. Virulence and pathogenicity of certain WNV strains has also been strongly linked to resistance to IFN [20]. For the interferon response activation, cellular pattern recognition receptors (PRRs) recognize pathogen associated molecular patterns including viral genomic RNA secondary structures. Protein PRRs involved in viral dsRNA recognition include the retinoic acid inducible gene-1 (RIG-I), melanoma differentiation associated gene 5 (MDA-5), double stranded RNA-activated protein kinase (PKR), 2′ 5′ -oligoadenylate synthetases (OAS) and adenosine deaminase acting on RNAs (ADARs) [21], [22]. The interferon response mediates its antiviral effect by up-regulating the transcription of interferon-stimulated genes (ISGs) leading to a significant increase in production of various antiviral effector proteins. The 2′ 5′ -oligoadenylate synthetases (OAS) are key ISG effector proteins that help to amplify innate immune response to viral infection [23]. The common mode of action of family members of nucleotidyl transferases is that, upon interaction with dsRNA, become activated to polymerize ATP into unusual oligoadenylate chains [2′-5′ (A)] where the 2′ carbon of ribose sugar of an adenosine mono-phosphate is linked to the 5′ carbon of the next [24], [25]. dsRNA binding results in a conformational change that properly orients an aspartic acid triad necessary for catalysis in the active site of OAS enzymes [26]. These oligoadenylate chains bind to and activate the endoribonuclease RNAse L, which destroys all single-stranded RNA including viral RNA, thereby attenuating viral protein production [27], [28].

Several lines of evidence suggest that OAS enzymes play a key role in the IFN response to WNV infection. The importance of OAS enzymes in the antiviral response against WNV can be inferred from the finding that RNase L limits WNV spread in mouse models [29]. Sangster *et al*. [30] demonstrated that OAS1 and other OAS isoforms are able to reduce flavivirus yields by 99%. Additionally, a single nucleotide polymorphism (SNP) in the *OAS1* gene acts as a host genetic risk factor for humans in WNV infection [31]. A second SNP in humans also established the OAS1 gene as a potential genetic risk factor in WNV infection and progression of the disease [32]. The antiviral effect of OAS proteins have also been demonstrated against picornavirus, which has a positive single stranded RNA genome similar to WNV [33]. Human OAS1 isotypes p42 and p46 have been shown in human cell lines to block, in a RNase L dependent manner, the viral replication of Dengue virus, which also belongs to family *Flaviviridae* [34]. Taken together, these results suggest that OAS1 may play a role in human antiviral response to WNV infection.

To date, no specific OAS1 recognition sites have been identified within the WNV RNA genome. Given the importance of OAS enzymes to restrict viral propagation via dsRNA binding, we sought to identify and analyze specific dsRNA regions of the WNV genome responsible for OAS1 activation. dsRNAs with stable secondary structure are ideal activators of OAS1. Regions including the 5′-UTR/initial coding regions and the 3′-UTR from members of the *Flaviviridae* family form conserved dsRNA stem loops that are necessary for the regulation of viral genome replication [9], [11]. We initiated studies do determine whether a specific RNA construct at the 5′-end of the WNV genome (SLI/II/III) could activate OAS1 enzymatic activity. In the current study, we demonstrate the *in vitro* activation of OAS1 by the SLI/II/III of WNV. Small-angle X-ray scattering experiments demonstrated that the WNV SLI/II/III adopts multiple conformations, including a subset that supports the predicted secondary structure. Truncations to the SLI/II/III were used to narrow the minimal region required for OAS1 activation, and mutations in the RNA binding site of OAS1 confirmed the specificity of the interaction [26], [35]. Taken together, the results presented suggest the biophysical basis for the regulation of OAS1 enzymatic activity by conserved secondary structural elements at the 5′-end of the WNV RNA genome.

## Materials and Methods

### Expression and purification of recombinant human OAS1

Expression of recombinant human OAS1 p42 isoform (transcript variant 2) as a fusion protein in BL21 DE3-RIL cells (Invitrogen, USA) and subsequent purification of the free protein from the cleaved N-terminal affinity tag (GNSHT: GB1, Nus A, Streptavidin, 6xHis and TEV protease site) was performed as described previously [36]. Point mutants of OAS1 (S162G, R195E, K199E) were generated using site-specific primers designed using the Quikchange primer design program (Agilent technologies, USA). The Quikchange kit (Agilent technologies, USA) was used to generate overexpression plasmids with the desired mutation. Plasmids were confirmed by sequencing and then expressed/purified in the same way as described for wild type OAS1 [36].

### In vitro transcription and purification of RNA

The SLI/II/III encompassing the 5′-UTR and initial coding region of WNV (NY99iso-1, nucleotides 1–146) was generated by *in vitro* transcription from a linearized plasmid using a non-denaturing column chromatography approach [37], [38]. RNA homogeneity was assessed by denaturing Tris borate-EDTA polyacrylamide gel electrophoresis with 8 M urea after mixing with an equal volume of 2 X denaturing loading buffer (95% Formamide, 18 mM EDTA, 0.01% Xylene Cyanol, 0.02% Bromophenol Blue) and heated at 95 °C for 5 minutes in 1 X TBE buffer (89 mM Tris Base, 89 mM Boric acid, 2 mM EDTA, pH 8.0). The RNA concentration was determined by spectrophotometry at 260 nm. The following truncations of the SLI/II/III region were also produced in an identical manner: SLI (1–73 with an additional 5′ G), SLII (73–110 with an additional 5′ G), SLIII (111–146), SLI/II (1–109 with an additional 5′ G), and SLII/III (73–146 with an additional 5′ G). A ssRNA (TCTCAAAGAAACACGTGCCGCTTACGCCCACAGTGTTCT) was transcribed/purified in an identical manner to serve as a negative control and to ensure that no unintended by-products of the transcription reaction were leading to activation.

### Analytical ultracentrifugation (AUC)

The sedimentation velocity (SV) experiment for OAS1 was performed using an Optima XL-I analytical ultracentrifuge with an An60-Ti rotor at 20.0 °C as described previously [39]. Standard 12 mm double sector cells were used where OAS1 [0.4, 0.6 and 0.8 mg/mL in 50 mM Tris, 100 mM NaCl and 1 mm DTT (pH 7.5)] and buffer were loaded in appropriate channels. SV data were collected at 7-minute intervals at 280 nm and 45,000 rpm using an absorption optical system. Data were analyzed using the SEDFIT program [40], [41] to obtain the sedimentation coefficients at each concentration (*s_20,b_*) which were then corrected to standard solvent conditions (*s_20,w_*) using the algorithm SEDNTERP [42]. The *s_20,w_* (S) values for individual concentrations were then extrapolated to infinite dilution to obtain *s^0^_20,w_* (S).

### Dynamic light scattering (DLS)

Dynamic light scattering data were collected using a Zetasizer Nano S system (Malvern instruments Ltd, Malvern, UK) as described previously [43]. A scattering angle of 173° was employed. Wild type OAS1 in 50 mM Tris (pH 7.5), 100 mM NaCl and 1 mM DTT was filtered using a 0.1 μm syringe filter (Millipore, USA) and subjected to DLS measurements at 20.0 °C at 3 different concentrations. Similarly, the DLS data for the WNV SLI/II/III was collected at a single concentration in 50 mM Tris (pH 7.0), 100 mM NaCl. The molecular weight of OAS1 was calculated using a version of the Svedberg equation adjusted to include the equivalent hydrodynamic radius *r_H_* in place of the translational diffusion coefficient:
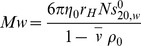
(1)


where 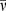 is the partial specific volume, *η_o_* is the solvent viscosity, *ρ_o_* is the solvent density and *N* is the Avogadro's number.

### Small angle X-ray scattering (SAXS)

SAXS data for proteins (wild type and mutants) and for the WNV SLI/II/III was collected using an in-house Rigaku instrument as described previously [44]. SAXS data for wild type OAS1, R195E and K199E mutants were collected at multiple concentrations (wild type: 3.1, 3.8, 4.5 and 5.2 mg/mL; R195E: 2.2, 2.6, 3.0 and 3.4 mg/mL; K199E: 2.3, 2.7, 3.1 and 3.5 mg/mL) in 50 mM Tris, 100 mM NaCl and 1 mM DTT at pH 7.5. SAXS data for the SLI/II/III (in 50 mM Tris, 100 mM NaCl and 20 mM MgCl_2_ at pH 7.0) were also collected at multiple concentrations (0.8 mg/mL, 1.6 mg/mL and 2.0 mg/mL). Primary data analysis was performed using the program PRIMUS [45], followed by estimation of the root mean square radius of gyration (*r_G_*) and the maximum particle dimension (*D_max_*) using the program GNOM [46]. *Ab initio* shape reconstruction of OAS1 WT was performed using the program DAMMIF, that utilizes simulated annealing protocol [47]. In addition to the *ab initio* shape determination, high-resolution structure of human OAS1 (PDB code: 4IG8 [26]) was used to reconstruct the solution conformation of OAS1 using the program BUNCH [48] as described earlier [49]. Twelve models using DAMMIF and ten models using BUNCH were generated which were then rotated, aligned and averaged using DAMAVER [50]. The program HYDROPRO [51] was employed to calculate solution properties such as hydrodynamic radius, radius of gyration and maximal particle dimension for each model calculated using SAXS data following a similar approach as outlined previously [44]. The input parameters included the density (1.0038 g/mL) and viscosity (0.01026 Poise) of buffer as well as partial specific volume of OAS1 (0.7424 mL/g), obtained from the program SEDNTERP [42]. The molecular weight of OAS1 was calculated from its amino-acid sequence using the protparam utility on Expasy server [52].

### Electrophoretic mobility shift assay (EMSA)

RNA (100 nM) was titrated with increasing concentration of OAS1, in 50 mM Tris, 100 mM NaCl (pH 7.5) buffer for EMSA experiments. The binding reaction was allowed to proceed for 10 minutes at room temperature (∼20.0°C) and the mixed with native loading buffer (to a final concentration of 0.02% bromophenol blue, 0.01% xylene cyanol FF and 1% glycerol in 1X TBE buffer) was added. Protein-RNA complex formation was analyzed on a Tris borate-EDTA poly-acrylamide gel (8%), and electrophoresis was performed at 65 V at ∼4.0°C in 0.5X TBE running buffer. Sybr gold (Invitrogen, USA) was used to visualize the RNA-containing species on the gel.

### OAS1 activity assay

OAS1 activity was measured using an established colorimetric assay that quantifies the amount of pyrophosphate (PPi) produced as a by-product of 2′ 5′-oligoadenylates formation by the active enzyme [36]. Briefly, reaction velocities (*V*) were calculated by linear regression within the linear range of time course in at least triplicate. The apparent dissociation constant (*K_app_*) and the maximum reaction velocity (*V_max_*) were determined using the following equation: *V* = *V_max_*/(1+(*K_app_*/[RNA])) [53]. OAS1 concentrations of 300 nM (for comparative activation assays) and 400 nM (for kinetic studies) were used as they fall within the concentration range from 50 to 400 nM previously been determined as optimal for kinetic analysis [36].

## Results

### Solution conformation of recombinant human OAS1

**Figure 2:**
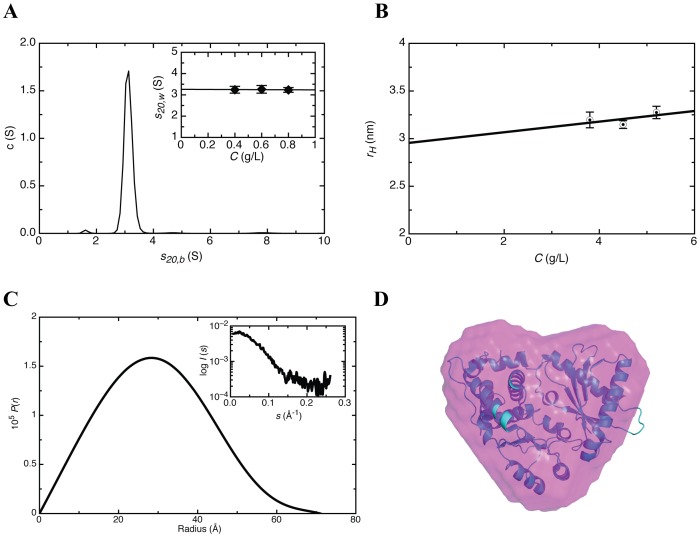
Recombinant human OAS1 adopts a globular fold. (**A**) Sedimentation velocity (SV) distribution analysis in terms of *c*(S) at 0.4 mg/mL. In-set is the resultant concentration dependence of the SV distribution. (**B**) Concentration dependence of hydrodynamic radius obtained from DLS measurements. (**C**) The pair distribution function versus particle radius obtained from the GNOM analysis. In-set is the merged scattering data obtained from multiple concentrations. (**D**) Superimposition of the human OAS1 (PDB 4IG8) high-resolution structure [26] on the *ab initio* model generated using DAMMIF on the data obtained from SAXS experiments on human OAS1.

In order to study the regulation of OAS1 activity by the SLI/II/III of the WNV genome, we first characterized the solution properties of the human recombinant protein to ensure homogeneity. Sedimentation velocity experiments using an analytical ultracentrifuge on purified OAS1 WT produced a single peak with a sedimentation coefficient value of 3.26±0.05 S (Svedberg units, S = 10^−13^ sec) suggesting that the protein is homogenous in mass and conformation (**Fig. 2A**). The homogeneity of OAS1 was further studied using DLS at multiple concentrations that provided the hydrodynamic radius (*r_H_*) of 3.0±0.3 nm for OAS1 (**Fig. 2B**). By taking the advantage of AUC and DLS data, an average molecular weight of 43.0 kDa was calculated for OAS1 that agrees well with the calculated molecular weight of 41.2 kDa. The results support the observation that OAS1 (p42 isotype) synthesized by cell free translation has been previous reported as monomeric [54]. A summary of all hydrodynamic properties for OAS1 is presented in **Table 1**.

**Table 1:** Experimental and predicted hydrodynamic parameters of OAS1 and SLI/II/III (error shown in parentheses).

	OAS1				SLI/II/III	
		HYDROPRORO				HYDROPRO
Parameter	Experimental	DAMMIF	BUNCH	4IG8^f^	Experimental	DAMMIF
*r_H_* (nm)^a^	3.0 (0.3)	3.13 (0.02)	3.05 (0.04)	2.90	5.1 (0.2)	5.00 (0.02)
*S°_20,w_* (S)^b^	3.26 (0.05)	3.13 (0.01)	3.23 (0.02)	3.16	ND	ND
*r_G_* (nm)^c^	2.28 (0.02)^d^	2.40 (0.01)	2.23 (0.01)	2.22	5.1 (0.1)	5.10 (0.01)
*D_max_* (nm)^c^	7.1^e^	6.90 (0.04)	6.80 (0.04)	6.6	16.0	16.8 (0.01)
?	-	0.9	1.0	-	-	1.0
NSD	-	0.52 (0.02)	0.36 (0.03)	-	-	1.10 (0.06)

a experimentally determined from DLS data.

b from AUC-SV data.

c from SAXS data.

d the *r_G_* values for R195E and K199E are 2.43 (0.11) nm and 2.40 (0.13) nm respectively.

e the *D_max_* values for R195E and K199E are 6.9 nm and 7.0 nm respectively.

f based on homology with high-resolution structure of human OAS1.

Next, the solution conformation of recombinant human OAS1 was determined. SAXS data were collected at multiple concentrations and merged to obtain a single output file (inset – **Fig. 2C**). A maximum particle dimension (*D_max_*) of 7.1 nm and a radius of gyration (*r_G_*) of 2.28±0.02 nm were obtained for OAS1 from the pair distribution function analysis (**Fig. 2C**). The *ab initio* shape reconstruction of OAS1 was performed and the goodness of fit parameter (χ value) of ∼0.9 was obtained for each individual model, signifying excellent agreement between the experimental scattering data and the calculated scattering data. The superimposed *ab initio* models provided an averaged model that was highly similar to each individual model in terms of shape as evidenced by normalized spatial discrepancy (NSD) parameter of 0.52±0.02 (**Fig. 2D**). The recently determined high-resolution structure of human OAS1 superimposed almost perfectly on the averaged *ab initio* model of OAS1 [26] (**Fig. 2D**). We additionally validated our *ab initio* modeling approach using the program BUNCH, that generated solution conformations of OAS1 based on existing high-resolution structural information that compared favorably with the *ab initio* models (**Table 1**). Furthermore, excellent agreement was observed between the experimentally determined hydrodynamic parameters from AUC, DLS and SAXS and parameters calculated from *ab initio* and BUNCH models of solution conformations (**Table 1**) using the program HYDROPRO. Hydrodynamic parameters calculated based on the previously published high-resolution structure of OAS1 are also in good agreement with the SAXS-derived models.

### Solution conformation of the SLI/II/III of WNV

**Figure 3:**
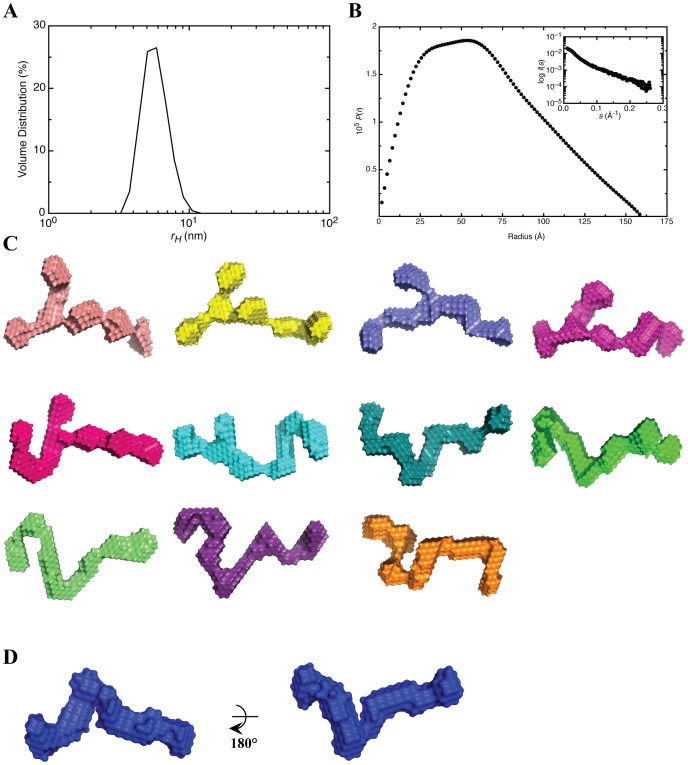
Solution conformations of the WNV SLI/II/III from SAXS. (**A**) Dynamic light scattering profile of SLI/II/III at 2 mg/mL. (**B**) Pair distribution function of SLI/II/III obtained from merged data of multiple concentrations. In-set is the merged SAXS data obtained from multiple concentrations. (**C**) Individual *ab*-*initio* models calculated from the SAXS data using DAMMIF program demonstrating two distinct subpopulations of the RNA molecule. (**D**) Averaged model of SLI/II/III obtained from individual models presented in **Fig. 3C**.

While RNAse probing experiments are consistent with the predicted secondary structure for the SLI/II/III of WNV, the three-dimensional structure of this RNA region is not known. We therefore *in vitro* transcribed WNV SLI/II/III (nucleotides 1–146, including the 5′-UTR and initial coding region) for the purpose of determining the solution structure by SAXS. Denaturing gel electrophoresis demonstrated a single band of appropriate size (data not shown), and DLS analysis confirmed that the sample was monodisperse with a *r* ***_H_*** of 5.1±0.2 nm (**Fig. 3A**). Raw SAXS data acquired at multiple concentrations were merged (**inset** **Fig. 3B**) and the pair distribution function analysis yielded *D_max_* of 16 nm and *r_G_* of 5.1±0.1 nm (**Fig. 3B** **,** **Table 1**). Interestingly, a number of alternative conformations of the SLI/II/III in solution were observed from the *ab initio* analysis of SAXS data with identical *D_max_* and *r_G_* values (**Fig. 3C**). This observation can likely be attributed to the underlying flexibility of the RNA molecule in solution. In a number of the determined solution conformations, three distinct protrusions are observed which may correspond to SLI (longer arm), SLII, and SLIII (shorter arm). **Fig. 3D** presents the averaged model obtained from superimposing the individual *ab initio* models calculated for SLI/II/III. Calculated hydrodynamic properties (*r_H_*, *r_G_* and *D_max_*) of the SLI/II/III were determined based on the *ab initio* models, and excellent agreement was found with the experimentally determined hydrodynamic properties (**Table 1**).

### The SLI/II/III of WNV interacts with and activates OAS1 *in vitro*

**Figure 4:**
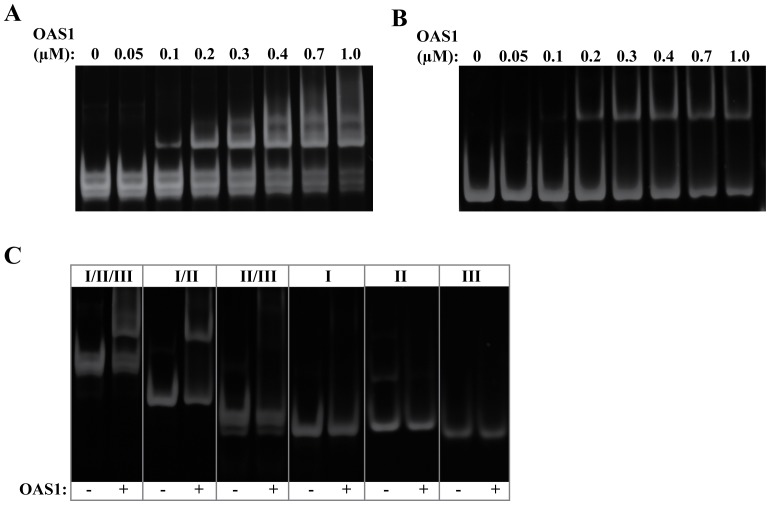
The WNV SLI/II/III forms a direct interaction with human OAS1. (**A**) EMSA for OAS1 (100 nM) binding to the SLI/II/III under non-denaturing conditions. (**B**) EMSA for OAS1 (100 nM) binding to SLI+II under non-denaturing conditions. (**C**) Non-denaturing gel electrophoresis of SLI/II/III truncations (100 nM) in the presence or absence of OAS1 (400 nM). In all cases, 8% native TBE gels were used and stained with Sybr Gold (Invitrogen, USA) to visualize RNA-containing species.

The SLI/II/III and initial coding region of the WNV genome is comprised of three double-stranded stem loops. We sought to determine whether this region of the genome could bind to and activate OAS1 *in vitro*. EMSA experiments of the SLI/II/III under non-denaturing conditions demonstrated that the RNA interacts with human OAS1 (**Fig. 4A**). With increasing concentrations of OAS1, the SLI/II/III is shifted into a higher molecular weight complex of increasing intensity. Interestingly, unbound SLI/II/III (**Fig. 4A** **, lane 1**) displays heterogeneity under native conditions consistent with the subspecies observed by SAXS. This heterogeneity is not observed under denaturing conditions (data not shown).

To identify the specific region(s) within the SLI/II/III that interacts with OAS1, we generated five different RNA molecules in addition to the SLI/II/III region that represent either two stem loops in combination (SLI/II, SLII/III) or individual stem loops (SLI, SLII and SLIII). We believe this truncation approach is feasible based on the predicted secondary structure and our observed solution conformation of the SLI/II/III. Complex formation with increasing OAS1 concentration was then performed for each RNA molecule in order to determine which secondary structural elements mediated the interaction. We observed significant complex formation that appeared as a high molecular weight species in the EMSA of SLI/II with OAS1 (**Fig. 4B**). At a constant RNA concentration of 100 nM, no detectable complex formation was observed with any individual stem loop (SLI, SLII, or SLIII or with the pairwise combination of SLII/III in the concentration range of 0 to 1 μM of OAS1 (**Fig. 4C**).

**Figure 5:**
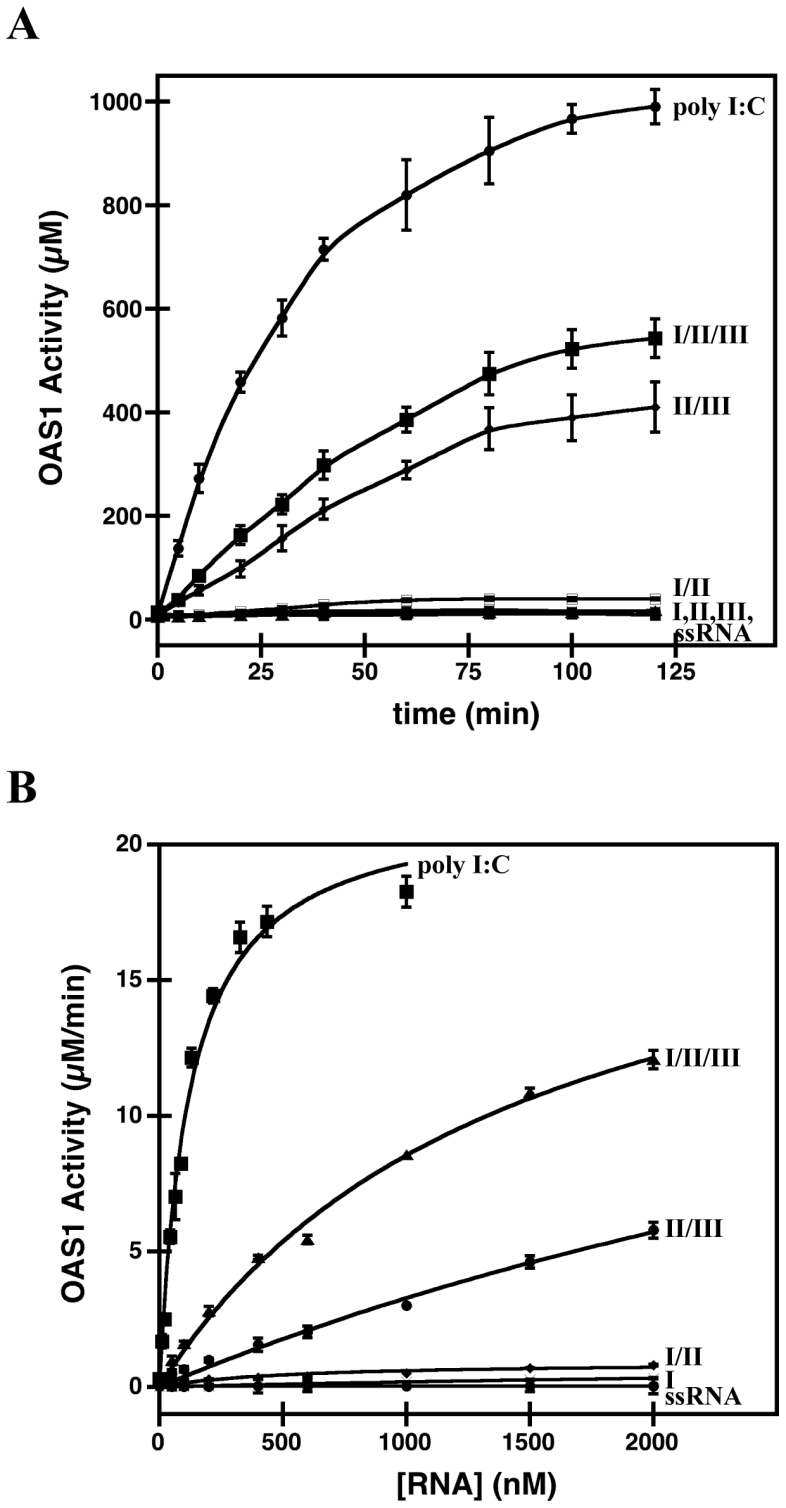
Catalytic activation of OAS1 by the SLI/II/III and its truncations. (**A**) Purified OAS1 (300 nM) and RNA (300 nM) were incubated in the presence of ATP (2 mM) and MgCl_2_ (5 mM) at 37°C, quenched at time points from 0–180 minutes, and 2′-5′(A) chain formation quantitated by PP_i_ detection. In all cases, errors represent the standard deviation from at least 3 replicates, and ssRNA represents a single-stranded negative control. (**B**) Enzymatic activity of OAS1 (400 nM) shown as a function of RNA concentration. Linear regression analysis of the initial velocity was used to determine OAS1 activity and the error in the analysis represented as error bars.

Upon establishing a direct interaction between the SLI/II/III of WNV and OAS1 *in vitro*, we were further interested to investigate whether this interaction leads to activation of OAS1 catalytic activity. A colorimetric assay was performed which correlates the detection of pyrophosphate (PPi) with the production of 2′-5′(A) chains. We prepared buffered reactions containing OAS1, ATP, and Mg^2+^ in the presence of SLI/II/III, polyinosinic-polycytidylic acid (poly I:C, a positive control synthetic dsRNA activator of OAS1), or a single-stranded RNA (ssRNA) negative control. The experiments were performed over a 120-minute period, followed by progressive measurement of PPi production (**Fig. 5A**). As expected, ssRNA negative control demonstrated no significant stimulation of OAS1 activity. The SLI/II/III activates OAS1 to a level that is approximately 60% of that achieved by poly I:C at all time points observed (on a per mass basis). However, direct quantitative comparison to the poly I:C control should be treated with caution given the extremely large size and heterogeneity of poly I:C (∼90 to 1400 kDa) relative to the WNV RNAs examined. Taken together, the structured SLI/II/III region of the WNV genome interacts with and activates OAS1 *in vitro*.

### SLI is dispensable for maximal OAS1 activation

To compare the ability of the stem loop regions of the SLI/II/III to activate OAS1, time courses monitoring of PPi production were performed and compared with the full-length SLI/II/III, poly I:C (positive control) and ssRNA (negative control). Remarkably, of all the SLI/II/III truncations, only SLII/III is capable of achieving activation level comparable to the full length SLI/II/III (**Fig. 5A**). Basal levels of PPi production, comparable to the negative control, were observed for SLI/II and each of the individual stem loops (SLI, SLII, and SLIII). Interestingly, the SLI/II construct that demonstrated high affinity complex formation with OAS1 did not stimulate catalytic activity, while SLII/III demonstrated potent activation despite a lack of detectable complex formation.

Due to the observed discrepancy between binding and activation, detailed dose-response experiments in which initial reaction velocities of OAS1-catalyzed PPi production were performed under increasing RNA concentrations for each of the SLI/II/III constructs (**Fig. 5B**). This approach allowed estimation of both the apparent dissociation constant (*K_app_*), a measure of affinity, and the maximum reaction velocity (*V_max_*), a measure of catalysis. **Table 2** summarizes the determined kinetic parameters for each RNA molecule, and includes a measure of the quality of the fit to the data (*R_fit_*). The SLI/II/III and SLII/III demonstrate potent stimulation of OAS1 activity (∼200-fold enhancement relative to the ssRNA negative control for both) despite the 4-fold higher affinity for OAS1 shown by SLI/II/III compared to the SLII/III. Despite having a *V_max_* value approaching that of the negative control, the SLI/II RNA demonstrated a nearly 3-fold increase in affinity relative to the SLI/II/III. None of the individual stem loop structures reveals appreciable stimulation of OAS1 catalytic activity, and only SLI demonstrates detectable affinity (4-fold lower affinity than the SLI/II/III). Together, the kinetic analysis supports a model where SLII/III is the minimal construct capable of OAS1 activation despite having a weak binding affinity for the enzyme.

**Table 2:** Comparison of kinetic parameters (*K_app_* and *V_max_*) of enzymatic activity of wild type OAS1 when activated by WNV SLI/II/III and its truncations.

RNA	*K*_app_	*V*_max_	R_fit_
	(nM)	(μM/min)	
I/II/III	1453±199	21±2	0.997
I/II	528±96	0.9±0.2	0.994
II/III	5775±452	22±2	0.995
I	5832±387	1.3±0.3	0.997
II	NA	NA	NA
III	NA	NA	NA
ssRNA	83±65	0.1±0.1	0.347
Poly I:C*	121±13	21.6±0.8	0.995

### Mutations to the dsRNA binding site disrupt activation of OAS1 by the SLI/II/III

**Figure 6:**
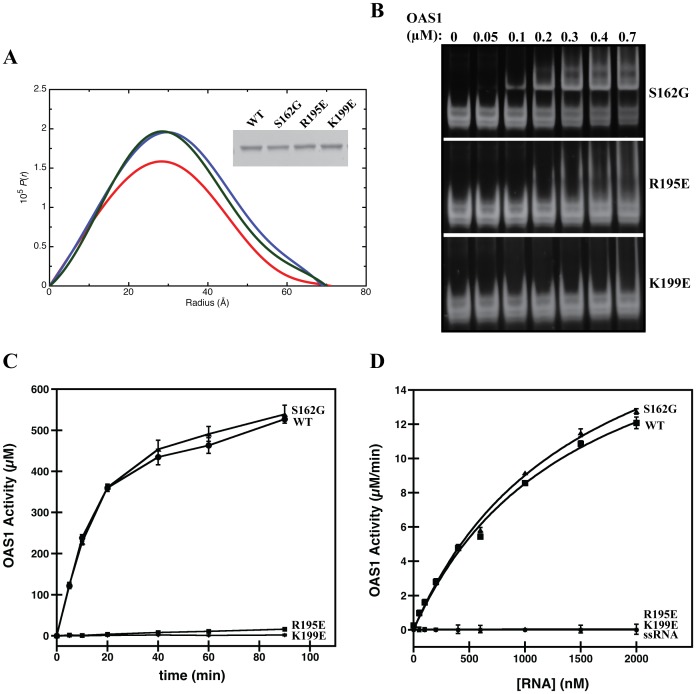
Analysis of OAS1 mutants. (**A**) Pair distribution function versus particle radius obtained from GNOM analysis for wild-type OAS1 (red), R195E (blue) and K199E (green). Inset, is a SDS-polyacrylamide gel presenting wild type and mutant OAS1s that suggests that all constructs have similar molecular weight. (**B**) EMSA for OAS1 and mutants (100 nM) binding to WNV SLI/II/III under non-denaturing conditions. (**C**) Reactions containing purified OAS1 or OAS1 mutants (300 nM) and RNA (300 nM) quenched at time points from 0–180 minutes followed by quantification of PP_i_ production. In all cases, errors represent the standard deviation from at least 3 replicates, and ssRNA represents a single-stranded negative control. (**D**) Enzymatic activity of OAS1 or OAS1 mutants (400 nM) shown as a function of RNA concentration. Linear regression analysis of the initial velocity was used to determine OAS1 activity and the error in the analysis represented as error bars.

To confirm that OAS1 activation is an RNA-mediated effect, the binding affinity and catalytic activity of two point mutants of human OAS1 (R195E and K199E) were investigated. Based on the human OAS1 structure, these mutations are in the positively charged dsRNA-binding groove on the enzyme face distal to the active site [26]. To verify that the mutations did not disrupt the native protein conformation, we performed SAXS experiments on OAS1 R195E and K199E. The resultant pair distribution function plots for wild type and mutants were nearly identical (**Fig. 6A**), and the determined *r_G_* and *D_max_* values were within error of the wild type results (data not shown). Therefore, we conclude that these mutations do not affect the solution conformation of OAS1.

As expected, higher molecular weight RNA-protein complexes were not observed in EMSAs of SLI/II/III with increasing concentrations of R195E or K199E OAS1 (**Fig. 6B**). We next investigated whether this loss of interaction had a similar impact on activation of OAS1 catalytic activity in the presence of SLI/II/III. Time course experiments following 2′-5′(A) synthesis by the mutants in the presence of SLI/II/III (**Fig. 6C**), SLII/III (data not shown), or poly I:C (data not shown) confirmed the expected attenuation of catalytic activity. For example, at the 90-minute time point, the R195E and K199E mutants demonstrated 3% and 0.4% of wild type activity respectively, in the presence of SLI/II/III. In an attempt to quantitate the impact, initial reaction velocities for wild type, R195E and K199E OAS1 were determined in a dsRNA dose response experiment using SLI/II/III as the activator (**Fig. 6D**). The low levels of catalytic activity demonstrated by the R195E and K199E mutants made accurate parameter determination impossible (**Table 3**). Therefore, we conclude that the SLI/II/III of WNV is mediating its effects through interaction with the previously reported dsRNA-binding site on OAS1 [26].

**Table 3:** Comparison of kinetic parameters (*K_app_* and *V_max_*) of enzymatic activity of wild type and mutant OAS1s when activated by the WNV SLI/II/III.

OAS1	*K*_app_	*V*_max_	R_fit_
	(nM)	(μM/min)	
WT	1453±199	21±2	0.997
S162G	1490±188	22±2	0.998
R195E	NA	NA	NA
K199E	NA	NA	NA

### Single nucleotide polymorphism in the OAS1 gene does not impede activation by dsRNA

The S162G mutation has been reported in a previous study as a very common single nucleotide polymorphism (SNP) in the OAS1 gene, and is more prevalent in WNV-susceptible individuals [55]. We therefore overexpressed and purified the mutant version of the protein, and investigated its ability to bind to and be activated by the SLI/II/III of WNV. Overall, no significant differences were observed for this mutant in terms of solution conformation (**Fig. 6A**), affinity for SLI/II/III (**Fig. 6B**), or ability to perform catalysis in the presence of dsRNA (**Fig. 6C, D**). These results are not surprising as this SNP is located on the face opposite the active site aspartic acids and does not mediate interactions with dsRNA [26], [55]. A comparison of the determined kinetic parameters is shown in **Table 3**.

## Discussion

OAS1 and other OAS isoforms play an important role in the recognition of viral dsRNA and subsequent amplification of the initial interferon-mediated innate immune response [24], [25], [27], [28]. Previous studies have linked SNPs in the OAS1 gene to susceptibility to WNV infection [31], [32]. The p42 isotype of OAS1 used our studies has been previously implicated to combat Dengue virus infection via an RNase L dependent pathway [34]. To best of our knowledge no direct enzymatic activation studies of OAS1 by regions of the WNV genome been previously performed. We therefore sought to investigate whether the WNV RNA genome served as a source for OAS1 activation. Our initial investigations focused on the SLI/II/III, based on its secondary structure that is conserved amongst *Flaviviridae* family members. We conclude that a direct interaction between the SLI/II/III of the WNV and OAS1 occurs *in vitro*, and therefore warrants further investigation in a cellular context.

Our study found that the SLI/II/III of WNV is a potent activator of OAS1 *in vitro*. The affinity of OAS1 for the SLI/II/III (*K_app_* of 1453±199 nM) is consistent with affinities for other short viral RNAs [36], and the maximum catalytic activity (*V_max_* of 21±2 μM/min) is within error of the positive control (synthetic poly I:C) (**Table 2**). Examination of various SLI/II/III truncations enabled a comprehensive analysis of RNA binding and activation potential to narrow down sufficient region(s). In tandem SLII/III are necessary for catalytic activation of OAS1 despite the requirement of SLI in conjunction with SLII for the highest affinity interaction. Stem loops SLII/III has a higher *K_app_* that shows weaker binding, which is supported by absence of any higher species in its EMSA in presence of OAS1 but also has a high *V_max_* comparable to poly I:C and SLI/II/III of WNV. This result supports previous observations that indicate that binding affinity does not necessarily correlate with the ability of an RNA molecule to stimulate the synthetase activity of OAS1; instead balance between sufficient affinity and stimulatory potential is the key [36], [53]. Overall, the SLI/II/III appears to achieve the best balance, but the construct lacking SLI is capable of achieving a similar maximum catalytic output to the full length SLI/II/III at higher RNA concentrations (**Table 2**).

We studied two point mutations in OAS1 within the RNA binding domain on the basic tract opposite the active site [26], [35]. These mutations did not impact the overall structure of OAS1 as determined by SAXS experiments (**Fig. 6A**). As expected, these mutants show no detectable affinity for WNV SLI/II/III, nor do they activate the catalytic activity of OAS1 (**Table 3**). This finding supports a previous study highlighting the importance of R195 and K199 residues in the proposed dsRNA binding site of OAS1 and confirms that dsRNA binding via these basic residues is crucial to the impact of WNV RNA on OAS1 activation [35]. Furthermore, this result highlights that while the RNA-protein interaction observed is in the μM range for the SLI/II/III, this affinity is more than sufficient for a dsRNA activator to activate OAS1.

Genome cyclization has been established as necessary for WNV genomic RNA replication, and involves a panhandle structure comprising long-range base pairing interactions between nucleotides in the SLI/II/III (5′-UAR in SLII and 5′-CS in SLIII and their complimentary nucleotides in 3′-UTR [13], [14], [17]. For genome cyclization to occur, both SLII and SLIII in the SLI/II/III unwind to form their new interactions. RNAse probing experiments have shown that while SLII and SLIII do adopt these long-range interactions, SLI of the SLI/II/III remains intact upon cyclization [11]. Our observations that the RNA construct comprising SLII/III is sufficient for maximum catalytic activation of OAS1 is particularly interesting in this context, as the replication-competent conformation of the SLI/II/III could potentially evade the OAS1-mediated innate immune response. Experiments are currently underway to investigate whether the panhandle structure attenuates activation of OAS1.

Although SAXS represents a low-resolution approach, the results presented are the first direct structural observation of the SLI/II/III from the WNV genome. The determined SAXS models of the SLI/II/III RNA suggest an inherently flexible molecule in solution. One subset of these conformations presented three distinct protrusions (that likely correspond to SLI, SLII, and SLIII respectively), whereas the remaining models lack domain resolution (**Fig. 3C**). The averaged solution conformation (**Fig. 3D**) has a relatively low NSD value (1.10±0.06), suggesting that these conformations are closely related to each other structurally. The most straightforward interpretation of these data is that a dynamic equilibrium between these conformations exists, and this idea is supported qualitatively by native gel electrophoresis where at least two distinct RNA conformations are observed. Given that genome cyclization with complimentary regions in the 3′-UTR would require unwinding of both SLII and SLIII in the SLI/II/III, it is enticing to speculate that our observed conformations represent the “structured” and “partially unwound” SLI/II/III conformations. As the structural features of the WNV 5′-UTR appear conserved amongst other *Flaviviridae* family members at the secondary structural level [11], [15], [16], the stem-loop recognition at the 5′-end observed in this study by OAS1 may possibly represent a general feature of OAS enzymes.
